# Impact of sertraline on serum concentration of CRP in hemodialysis patients with depression

**DOI:** 10.15171/jrip.2017.12

**Published:** 2016-08-26

**Authors:** Narges Sadat Zahed, Marjan Sharifi, Mahdi Karimi, Hajar Nikbakht

**Affiliations:** Loghman Hakim Clinical Research Development Center, Shahid Beheshti University of Medical Sciences, Tehran, Iran

**Keywords:** Depression, Hemodialysis, Inflammation, CRP, Sertraline, BDI-II score

## Abstract

**Introduction:** Depression is the most prevalent psychological problem among hemodialysis (HD) patients. Inflammatory factors have been reported to play an important role in the pathogenesis of depression. The association between depression and inflammatory factors was established in chronic kidney disease (CKD) patients. Sertraline, a selective serotonin reuptake inhibitor (SSRI) antidepressant, decreases serum levels of inflammatory factors in patients with depression.

**Objectives:** This study was designed to assess the effect of sertraline on serum concentration of C-reactive protein (CRP), hemoglobin and albumin of depressed hemodialysis (HD) patients.

**Patients and Methods:** During a clinical trail, 35 depressed HD patients, and CRP >5 were allocated to receive sertraline for 12 weeks. Patients’ depression was assessed using Beck depression inventory second edition (BDI-II) biochemical parameters (hemoglobin, serum albumin, etc) and CRP levels were measured at baseline and at weeks 4, 8 and 12 of the study. BDI-II score was evaluated before and after 12 weeks treatment with sertraline.

**Results:** Sertraline significantly improved depression symptoms in HD patients. At the end of the study, BDI-II scores significantly changed from baseline (*P*=0.001) and serum levels of CRP significantly decreased at week 12 of initiation of the study (*P*=0.001). However, the concentration of hemoglobin and serum albumin concentration and weight was not changed significantly (*P*=0.995 and *P*=0.328, respectively).

**Conclusion:** Sertraline significantly decreases CRP levels and can be a promising strategy to reduce the systemic inflammation and to treat depression in HD patients.

**Implication for health policy/practice/research/medical education:**

Depression is the most prevalent psychological problem among hemodialysis (HD) patients. Inflammatory factors have been reported to play an important role in the pathogenesis of depression. Patients’ depression was assessed using Beck depression inventory second edition (BDI-II) biochemical parameters and C-reactive protein (CRP) levels were measured at baseline and at weeks 4, 8 and 12 of the study. This study was designed to assess the effect of sertraline on serum concentrations of CRP, hemoglobin and albumin of depressed HD patients. Sertraline significantly decreases CRP levels and can be a promising strategy to reduce the systemic inflammation and to treat depression in HD patients.

## Introduction

Chronic kidney disease (CKD) is a range of different pathophysiologic processes lead to abnormal renal function and decline in glomerular filtration rate. CKD is an inflammatory condition which is associated with an increasing in acute phase reactants, such as circulating inflammatory cytokines and C-reactive protein (CRP) and also decreasing in negative acute phase reactants, such as serum albumin and fetuin (1).

CRP is a sensitive marker of inflammation and tissue damage. CRP production is under the IL6 control with a fast rise by pathological stimuli and after pathological agents subsidence, CRP levels quickly drop (2). Depression is a common problem in patients with end-stage renal disease (ESRD) undergoing hemodialysis (HD). Depression prevalence in the general population is 7%-10% while in studies among CKD patients is 20% (3,4).

Chronic inflammation is common in patients with ESRD undergoing chronic HD and associated with atherosclerosis, cardiovascular diseases, malnutrition and increased mortality rate (4). It is established that inflammatory factors such as CRP and IL6 rise in ESRD patients (5). Chronic inflammation plays an important role in the pathophysiology of depression (6). Some previous studies detected increasing inflammatory markers such as CRP, interleukins, ferritin and erythrocyte sedimentation rate (ESR) in CKD patients, leading to deterioration of depression and subsequent malnutrition, atherosclerosis, cardiovascular morbidity and mortality in ESRD patients (7,8). Patients with ESRD and depression, have higher levels of CRP and serum ferritin levels. Whereas hemoglobin, hematocrit and albumin were lower among ESRD patients without major depression (9). It seems that reduction in inflammatory factors in ESRD patients could reduce depression and subsequently decreases cardiovascular events and mortality rate (10).

It is suggested that antidepressant drugs which have also anti-inflammatory effects, may lead to decline the levels of inflammatory markers (11,12).

According to studies, the best antidepressant medicine for the treatment of depression

in patients with ESRD is sertraline. In addition to treating depression, sertraline is capable to reduce pro-inflammatory cytokines and CRP (13,14).

In most studies BDI is used to screen patients in which 21 items is used to measure depressive symptoms and total score is ranged from 0 to 63. In the general population depression inventory second edition (BDI) scores >10 need treatment while in CKD patients BDI score > 14 is considered for medical therapy (15).

Sertraline as the first line treatment is well tolerable in patients with ESRD and does not need dose adjustment (16,17). In the general population treatment with sertraline reduced the pro-inflammatory cytokines like IL2, IL4 and increased the anti-inflammatory cytokines (18).

## Objectives

In this study, we aimed to assess the impact of sertraline, as a selective serotonin reuptake inhibitor (SSRI) antidepressant, on inflammatory markers among HD patients with major depression.

## Patients and Methods

In this investigation, 51 depressed patients fulfilled the inclusion criteria. However, according to drug side effects, death and hospital admission, 43 patients were investigated finally.

The inclusion criteria were as follows: CRP> 5 mg/dL, age older than 18 years, undergoing dialysis for at least 3 months, and BDI score >14.

Patients were excluded if they had active infections, malignancy, autoimmune disease, severe mental illness or cognitive disorders, sertraline hypersensitivity, treatment with NSAIDs, antibiotics or steroids one month before the study.

All of our participants needing medical treatment had BDI score>14 because ethical committee did not permit to have a placebo group in the present study.

Patients were under treatment for 12 weeks. Sertraline was administered 50 mg/day as an initial dose. Each two weeks, participants were visited and the drug dose could increase if necessary until reaching maximum dose of 200 mg/day.

At the beginning of the study, CRP, complete blood count (CBC), albumin, hemoglobin and patients’ weight were evaluated. After 12 weeks of treatment, patients’ depression was evaluated by Beak II.

### Ethical issues

The research followed the tenets of the Declaration of Helsinki; informed consent was obtained; and the research was approved by the ethical committee of Shahid Beheshti University of Medical Sciences.

### Statistical analysis

Data was analyzed using SPSS version 18 (SPSS Inc., Chicago, IL, USA). Results are shown as mean ± SD or median for normally or not-normally distributed continuous variable and number or percentages in the case of nominal variables. To evaluate before and after changes paired *t-*test was used. *P*<0.05 was considered as significant.

## Results

Baseline parameters like demographic characteristics dialysis duration and etiology of ESRD are shown in Table 1.

**Table 1:** Patients’ characteristics

**Parameter**	**Value**
Age (years)	
Median (range)	60.8 ± 13.8
Median (range)	62 (27-79)
Sex	
Female‏	21 (60%)
Male	14 (40%)
Dialysis duration	
Mean‏ ± SD	5.76 ± 3.4
Median (range)	4 (1-18)
Etiology of ESRD	
HTN	9 (25.8%)
Diabetes	18 (51.4%)
Other causes	8 (22.8%)

The mean of BDI-II score at week 0 was 21.4 ± 6.98 and after 12 weeks of treatment decreased to 13.31 ± 5.59 which was statistically significant (*P*=0.005).

**Figure 1:**
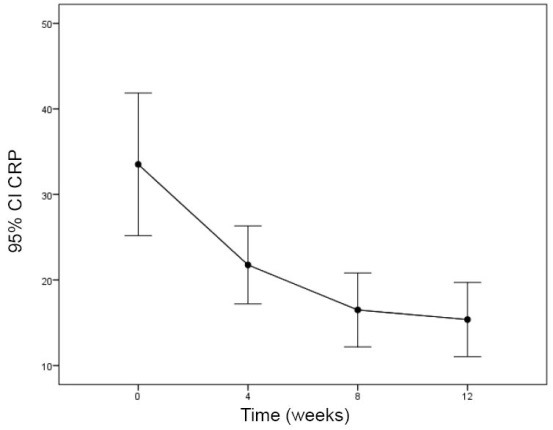
Effect of sertralin on CRP after 12 weeks of treatment.

**Figure 2:**
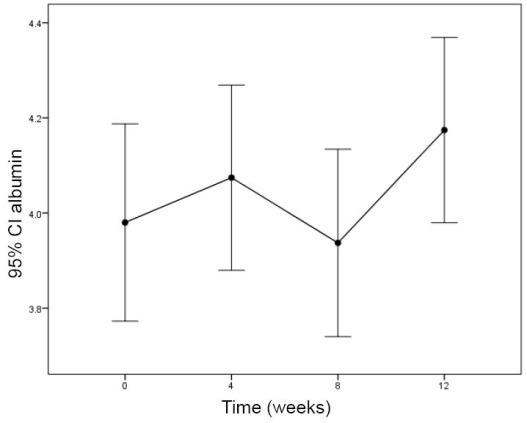
Effect of sertralin on albumin after 12 weeks of treatment

**Figure 3:**
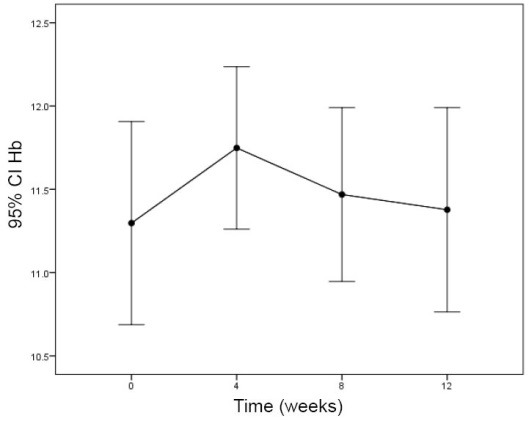
Effect of sertralin on Hb after 12 weeks of treatment.

The effect of sertraline on CRP after 12 weeks was statistically significant (Figure 1; *P*=0.001) whereas, albumin and hemoglobin did not change significantly (Table 2; Figures 2 and 3).

**Table 2:** Parameters mean changes

**Parameters**	**Week 0**	**Week 4**	**Week 8**	**Week 12**	**P value**
weight (kg)	62.7 ± 13	62.3 ± 12	62.5 ± 12.9	62.4 ± 12.9	0.569
CRP (mg/dL)	33.5 ± 24.2	21.8 ± 13.3	16.5 ± 12.6	15.4 ± 12.6	0.000
Albumin (g/dL)	3.09 ± 0.86	4.07 ± 0.57	3.94 ± 5.7	4.17 ± 0.57	0.328
WBC(10^3^/mm^3^)	7.47 ± 4.8	7.33 ± 3.1	7.6 ± 3.04	7.26 ± 2.7	0.982
Hb (g/dL)	11.3 ± 1.7	11.7 ± 1.4	11.4 ± 1.5	11.3 ± 1.7	0.995
PLT(10^3^/mm^3^)	187 ± 77	183 ± 69	201 ± 78	202 ± 87	0.713
Na (mEq/L)	139 ± 4	138 ± 3	137 ± 6	137 ± 3	0.133
K (mEq/L)	5.5 ± 0.8	5.4 ± 0.8	5.7 ± 1.1	5.8 ± 1.1	0.462

Abbreviations: CRP, C-reactive protein; WBC, white blood cell; Hb, hemoglobin; PLT, platelet; Na, sodium; K, potassium

## Discussion

This study has evaluated the effect of sertraline on inflammation markers in HD patients.

According to results, sertraline had anti-inflammatory effect and decreased the CRP as an inflammatory marker. The study conducted by Dashti et al on 300 HD patients showed high levels of CRP, and low albumin levels were highly prevalent among ESRD patients and resulted in morbidity and mortality (6).

In the current study, we found CRP >5 mg/dL in 57 % of participants, which was as same as previous studies, while hypoalbuminemia was not found in our study. Kalender et al showed accompany of high levels of CRP and decrease in albumin and hemoglobin in HD patients with depression (16).

In our study, we found depression among 51 patients with high CRP levels. Although previous studies (6,19) evaluated the impact of sertraline on CRP levels in comparison with the placebo group, in our study, according to BDI score >14, it was not permitted by the ethics committee to consider the placebo group without treatment.

Additionally, Dogan et al (15) found a significant correlation of depression score and levels of CRP whereas in our study, we had patients with very high CRP levels whose depression score was low.

In one study, CRP levels was measured before and after treatment, while we concluded that antidepressant drugs, not only treated depression, but also reduced inflammatory markers (20).

Likewise, in CAST study sertraline was administered as an antidepressant drug in CKD patients which could improve nutrition, quality of life and depression signs (21).

The present clinical trial evaluated the impact of the sertraline effect (50 mg/day).

Some adverse events like headache and dizziness were observed in the fewest number of participants, which resolved by decreasing the sertraline dose and only two patients were refused to complete the study.

We also found the significant decrease in CRP levels after 4 weeks of treatment. In line with our results, Pizz et al showed decline in CRP levels after treatment with sertraline among patients with cardiovascular disease too (19). However, in a study on patients with coronary heart disease and comorbid major depression, the increase in CRP levels by sertraline therapy was found (20).

Tuglu et al (21) and O’Brien et al (17) suggested significant decrease in CRP plasma levels by SSRI therapy in depressive population.

In this study, we did not find significant changes in albumin levels after 4, 8 and 12 weeks of treatment in HD patients, whereas Koo et al showed treatment with paroxetine leading to increase in albumin levels (22).

Albumin is a protein which is related to diet and oral intake. Inflammatory markers such as cytokines result in activating protein catabolism and malnutrition. It seems that, treatment with antidepressant drugs improves oral intake and also declines in protein catabolism. In a study by Lee et al depression led to insufficient oral intake in HD patients and after treatment of depressive disease weight gain was detected among studied population (23).

Despite the improved appetite in our study, we did not find the increase in serum albumin value and an improvement of weight gain. This different result may be due to differences of the patients’ diet.

Significant changes in hemoglobin levels was not detected. Our findings was in line with Kalantar-Zadeh et al (24). We could not control and set the erythropoietin dose weekly, thus, hemoglobin levels did not show significant changes in this study.

## Conclusion

Sertraline is an SSRI which does not need dose adjustment in ESRD or HD patients and also has few tolerable adverse events. Administration of sertraline may lead to a decrease in inflammatory markers and also improvement of depressive signs leads to better quality of life, reduced morbidity and finally mortality among HD patients. Therefore, more studies are needed to determine the appropriate doses.

## Limitations of the study

The limitations of our study were the small sample size and short duration of the investigation.

## Acknowledgments

This study was presented as a poster presentation at International Congress of Nephrology & Urology on June 12, 2015 in Tehran.

## Authors’ contribution

NSZ has designed the study and observed accuracy and validity of study protocols. MS collected data and followed the studies objects. HN wrote the article and edited the manuscript.

## Conflicts of interest

The authors declare no conflict of interest.

## Ethical considerations

Ethical issues (including plagiarism, data fabrication, double publication) have been completely observed by the authors.

## Funding/Support

This article is extracted from internal medicine residential thesis of Marjan Sharifi (Thesis #296).
